# Characterization of bipolar plates manufactured with various Pb/C ratios for unitized regenerative fuel cell system

**DOI:** 10.3389/fchem.2023.1178787

**Published:** 2023-05-04

**Authors:** Chanmin Jo, Yoongu Lim, Dae Jun Moon, Seungryul Yoo, Dong Chan Seok, Seon Yeop Jung, Seunghun Jung, Ho-Young Jung, Uk Sim

**Affiliations:** ^1^ Hydrogen Energy Technology Laboratory, Korea Institute of Energy Technology (KENTECH), Naju, Republic of Korea; ^2^ Institute of Plasma Technology, Korea Institute of Fusion Energy (KFE), Gunsan, Jeollabuk-do, Republic of Korea; ^3^ Department of Chemical Engineering, Dankook University, Yongin-si, Gyeonggi-do, Republic of Korea; ^4^ Department of Mechanical Engineering, Chonnam National University, Gwangju, Republic of Korea; ^5^ Department of Environment and Energy Engineering, Chonnam National University, Gwangju, Republic of Korea; ^6^ Center for Energy Storage System, Chonnam National University, Gwangju, Republic of Korea

**Keywords:** metal-carbon, composite materials, Pb/C, bipolar plate, unitized regenerative fuel cell (URFC)

## Abstract

The weight reduction of the bipolar plate (BP) is essential for commercializing unitized regenerative fuel cells (URFCs). In order to lighten the weight of the bipolar plate, we have used Pb/C composite powder as a cost-effective primary material, which is a mixture of low-density graphite and lead. Further, varied lead-carbon weight ratios (1: 8, 1:4, 1:1, 4:1, and 8:1) were investigated for fabricating the bipolar plate by hot-pressing process adding styrene-butadiene rubber (SBR) as a binder. The specific surface area, porosity, and microstructure characteristics corresponding to the varied lead-graphite ratio of the prepared bipolar plates were studied. The relative difference in conductivity upon the compressibility of the plates is also examined. Finally, the wettability and electrochemical properties of the prepared bipolar plates were evaluated through water contact angle and cyclic voltammetry analysis.

## 1 Introduction

In this contemporary world, energy usage is highly indispensable, but the associated undesirable outcomes in the process of energy production and consumption, such as the greenhouse effect, pollution, carbon dioxide emission, and fuel resource depletion, are steadily increasing. Hence, there is a need for the development and application of environmental-friendly renewable energy (Wang et al., 2015; De Luna et al., 2019; Hussain et al., 2020). Accordingly, effectual research is being conducted worldwide on hydrogen energy that has high energy density and can be continuously produced without carbon dioxide emission (Ozawa et al., 2019; Yue et al., 2021).

In order to utilize such hydrogen energy, water electrolyzer (Gago et al., 2016; An et al., 2019; Klose et al., 2020; Janani et al., 2021) and fuel cell (Du et al., 2016; Ren et al., 2022; Reshetenko et al., 2022) technologies are attracting attention. Hydrogen and oxygen are produced from water through a water electrolysis device using an exchange membrane, whereas electricity is produced through a chemical reaction between hydrogen and oxygen using a fuel cell. Moreover, research interest in a hybrid system called regenerative fuel cells (RFCs) that can produce hydrogen and electricity by applying water electrolysis and fuel cell technology is increasing (Lim et al., 2020; Rubio-Garcia et al., 2020). However, the RFC is configured to combine both the water electrolyzer and the fuel cell systems. Hence the volume and weight of the RFC as a whole increase due to the volume and weight of each. Thus, an improvised single-device URFC integrating a water electrolyzer and a fuel cell is designed to solve the above issue by switching between both modes (Peng et al., 2020; Zhang et al., 2022). It is a system that has been structurally improved so that the two modes can be used selectively, and research on this is ongoing. Although the volume and weight were reduced by integrating the two devices, there are certain restraints to reducing the weight through this. Hence further research on the weight reduction of the URFC system that requires multiple stacks becomes necessary (Paul and Andrews, 2017). The stacked system used in water electrolyzers and fuel cells consists of a membrane-electrode assembly (MEA) consisting of a bipolar plate, a catalytic electrode, a membrane, and an end plate (Wu et al., 2020). A bipolar plate is inserted between each MEA, and each side of the bipolar plate is in contact with the cathode and anode of the MEA (Gabbasa et al., 2014). Therefore, structurally, the number of bipolar plates also increases according to the number of MEA stacks. A sufficient number of MEA stacks is required for the high power output of the system (Devrim et al., 2015). That is, the weight of the entire system inevitably increases for high power output, and a significant portion of the weight is occupied by the weight of the bipolar plate. Accordingly, since the weight of the entire system increases in proportion to the number, material, size, *etc.*, of the separators, it has limitations in being used in transport systems and in areas requiring many stacks, lightweight or miniaturization (Kolahdooz et al., 2017; Lim et al., 2019; Li et al., 2021).

Among the factors that affect weight, the material constituting the bipolar plate is the most basic element, and to manufacture the bipolar plate efficiently, the economic feasibility of the material, electrical conductivity, weight, corrosion resistance, strength, and required process level are important considerations (Karacan et al., 2020; Wu et al., 2021; Xiong et al., 2021). The main materials used for the bipolar plate are classified into metal, carbon, and composite materials. Among metal materials, stainless steel (SS), Ti, and Al are mainly used for bipolar plates. Chen et al. performed a conductive polymer coating utilizing carbon powder, poly-pyrrole (PPy), and polydopamine (PDA) on the surface of 304 SS. The formed PPy/C-PDA coating layer acted as a physical barrier and showed a protective effect against corrosive substances, improved interfacial contact resistance, and finally improved corrosion resistance (Chen et al., 2020).

Shi et al. showed improved electrical conductivity and corrosion resistance by forming a TiC layer on the surface of a Ti bipolar plate (Shi et al., 2020). Tsai et al. increased surface hydrophobicity and improved corrosion resistance by forming an Au-PTFE coating layer on the surface of an Al bipolar plate (Tsai et al., 2017). Sadhasivam et al. fabricated a lead-based bipolar plate and showed the possibility of applying a cost-effective Pb bipolar plate (Sadhasivam et al., 2020). Pan et al. reported improved corrosion resistance and excellent coating adhesion through CrN coating on Fe-Cr bipolar plates (Pan et al., 2014). Research on bipolar plates of carbon materials has also been conducted. Yan et al. fabricated a graphite bipolar plate, showing its applicability to 130 stacks level PEMFC (Yan et al., 2006). However, there are certain hinges with the strength of pristine carbon materials, so research is being conducted on composite materials used with polymer materials that act as conductive binders. Yao et al. fabricated a bipolar plate with a mixture of graphite and polymer resin and showed uniform performance in terms of thickness, corrosion resistance, mechanical strength, and electrical and thermal conductivity (Yao et al., 2017). Choi et al. fabricated a thin bipolar plate by mixing carbon fiber and resin and showed electrical conductivity, mechanical strength, and gas permeability compared to carbon BP and metal BP (Choi et al., 2021). Adloo et al. compared bipolar plates made of various carbon materials (graphite, graphene, and highly structured nano-carbon black (HSNCB)) and polypropylene at various ratios. Among them, a bipolar plate made of 23% polypropylene–65% graphite–7% HSNCB–5% polypropylene-maleic anhydride (pp-MAH) exhibited excellent electrical conductivity and flexural strength (Adloo et al., 2016). In order to reduce the weight of bipolar plates, it is essential to use lightweight carbon materials due to their low density. However, carbon alone cannot satisfy all the characteristic requirements of bipolar plates. To solve this problem, metal-carbon composites are being researched to improve the strength, corrosion resistance and conductivity of carbon. Soleimani Alavijeh et al. fabricated a bipolar plate using epoxy, graphite, and nano-copper. Nano-copper was utilized as a filler to increase the strength through proper proportions, and the presence of nano-copper resulted in higher conductivity (Soleimani Alavijeh et al., 2019). Among the various metal candidates, lead is utilized in acid batteries because it is inexpensive and has good conductivity and corrosion resistance, and these properties make it a promising material for bipolar plates for URFCs that need to apply high voltages and operate in the water electrolysis mode, which requires corrosion resistance (Jung et al., 2010; May et al., 2018).

In this study, a bipolar plate using a Pb/C composite material was manufactured by using a carbon material with excellent light weight and conductivity and lead, a metal material with excellent strength and corrosion resistance, to produce a positive plate. The results of the physical and electrochemical properties of the bipolar plates manufactured with various Pb/C ratios suggested the optimal manufacturing conditions of the Pb/C bipolar plates for the URFC system.

## 2 Experimental methods

### 2.1 Materials

Graphite (<20 μm) and lead (325 mesh) were purchased from Sigma-Aldrich. Styrene-butadiene rubber (SBR) was purchased from MTI Korea, Republic of Korea. Alumina ball (99.5%, 10 ϕ) was purchased from Labkom, Republic of Korea.

### 2.2 Fabrication of bipolar plates

Initially, Pb/C powder (weight ratio 8:1, 4:1, 1:1, 1:4, 1:8) in quantities of 60 g, 70 g, 140 g, 210 g, and 360 g were prepared and mixed with 45 g of SBR. The above mixture of Pb/C and binder is transferred to a metal mold of size 75 × 75 mm, which is subjected to a hot press (QM900M, QMESIS, Republic of Korea.). Hot pressing is carried out in three steps. At first, the molds are maintained at 70° for 40 min under 10 MPa, then at 140° for 40 min under 20 MPa, and finally, at 140° for 1 h under 30 MPa. Once the process is done, it is cooled and de-molded to obtain the desired bipolar plates.

### 2.3 Physicochemical characterizations

The morphology and surface chemistry of the as-obtained Pb/C bipolar plates were studied by scanning electron microscope (SEM, Gemini 500, ZEISS, Germany) and energy dispersive X-ray spectroscopy (EDS, Oxford Instruments, UK), respectively. The powder X-ray diffraction (XRD, Rint 1,000, Rigaku, Japan) patterns of Pb/C bipolar plates were obtained with Cu Kα radiation (*λ* = 1.5418 Å). X-ray photoelectron spectrum was attained through X-ray photoelectron spectroscopy (XPS, Multilab 2000; UK). The contact angle of Pb/C bipolar plates was determined through a contact angle meter (Phoenix 300, SEO, Republic of Korea). The specific surface area and the total pore volumes of the synthesized bipolar plates were measured with BET, Belsorp mini II (BEL, Japan). Powder resistivity was measured by powder resistivity measurement system (Hantech, Republic of Korea). 2.6 g of each prepared Pb/C powder was placed in a measuring mold and measured using a powder resistivity meter. The pressure range was set from 20 MPa to 200 MPa, and the resistivity, conductivity, and thickness were measured at 20 MPa intervals.

### 2.4 Electrochemical measurements

Electrochemical characterization of the Pb/C bipolar plate was carried out using potentiostat equipment (Bio-Logic SP150 instrumentation) at room temperature in a three-electrode cell configuration. 0.5 M H_2_SO_4_ solution was used as an electrolyte. The platinum (Pt) wire and Ag/AgCl reference electrodes were used as counter and reference electrodes. The synthesized plates were cut into 2 × 1 cm^2^ and were used as the working electrodes.

## 3 Results and discussion

### 3.1 Characterization of Pb/C bipolar plates

**FIGURE 1:**
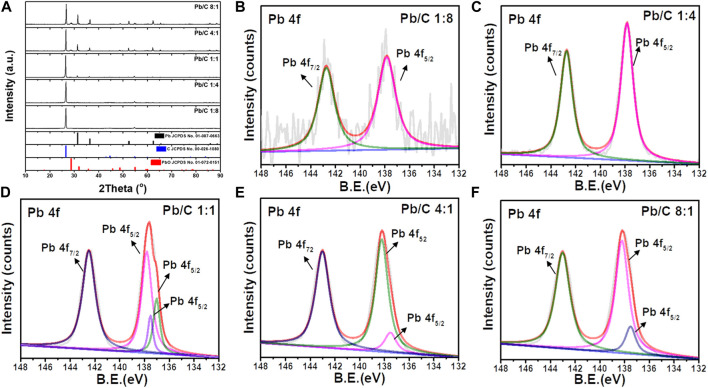
**(A)** X-ray diffraction patterns for different ratio of Pb/C powder, XPS spectra of Pb/C powder **(B)** 1:8, **(C)** 1:4, **(D)** 1:1, **(E)** 4:1 and **(F)** 8:1 ratios.

X-ray diffraction (XRD) patterns of Pb/C bipolar plates with a different lead-carbon ratio ranging from 8:1 to 1:8 is shown in Figure 1. The XRD peaks were analyzed with the reference patterns of Pb (JCPDS No. 01-087-0663) and C (JCPDS No. 00-026-1,080) with the addition of PbO (JCPDS No. 01-072-0151) which may result from partial oxidation during the synthesis process. Throughout the different ratios of Pb and C, the high intensity peaks at 26.7° can be indicated as the (002) plane of carbon. Although in 1:4 and 1:8 ratios of Pb and C, the synthesized plates show a low indication of Pb due to the high content of C, the Pb peaks were distinguished at 32.1, 52.4, and 62.3° at the higher ratio from 1:1, 4:1, and 8:1. These peaks correspond to the (111), (220), and (311) planes of Pb, respectively. Furthermore, as the Pb content was increased, the diffraction peaks of PbO were detected at 28.2, 48.8, and 54.8°, which corresponds with (111), (112), and (211) planes of PbO, respectively. The formation of PbO can be due to the high content of Pb, where the excessive Pb was oxidized.

**FIGURE 2:**
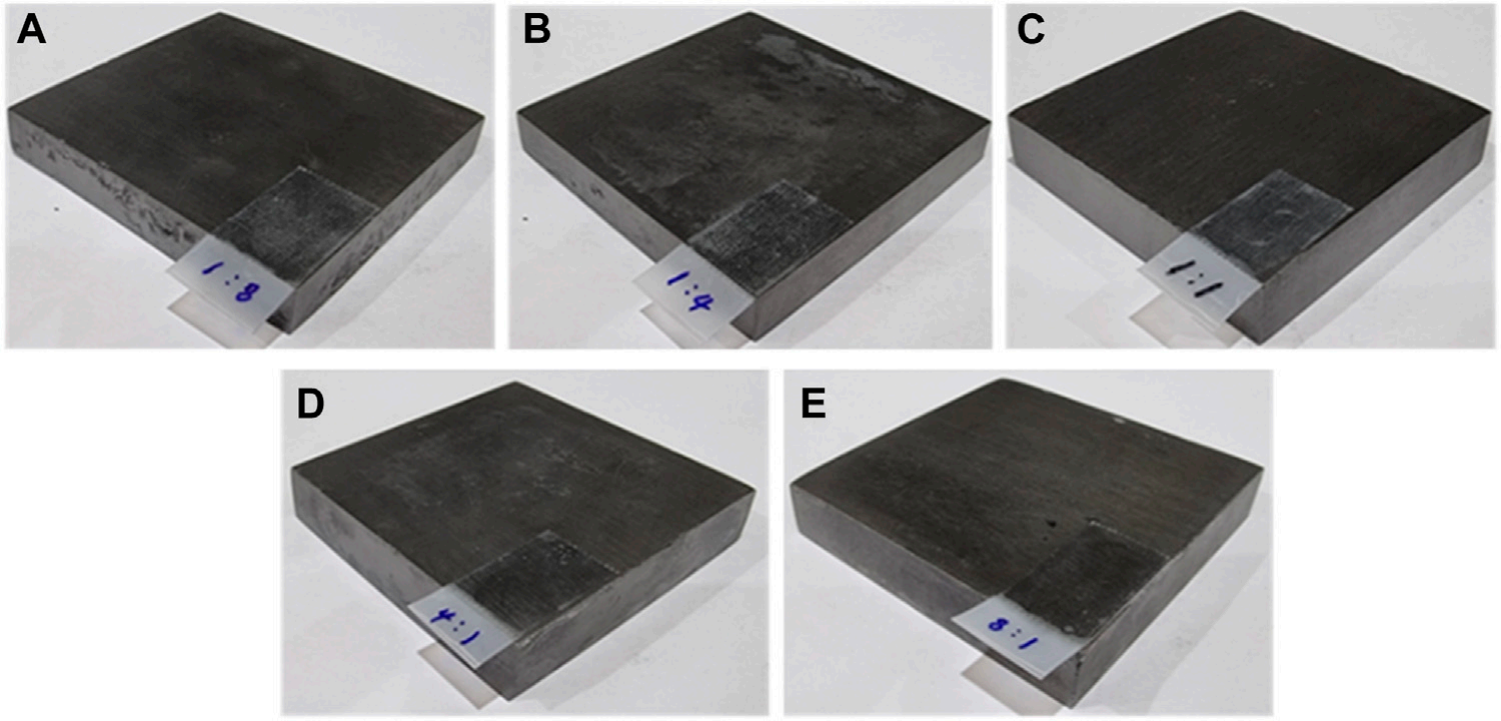
Fabricated Pb/C bipolar plates with **(A)** 1:8, **(B)** 1:4, **(C)** 1:1, **(D)** 4:1 and **(E)** 8:1 ratios.

**FIGURE 3:**
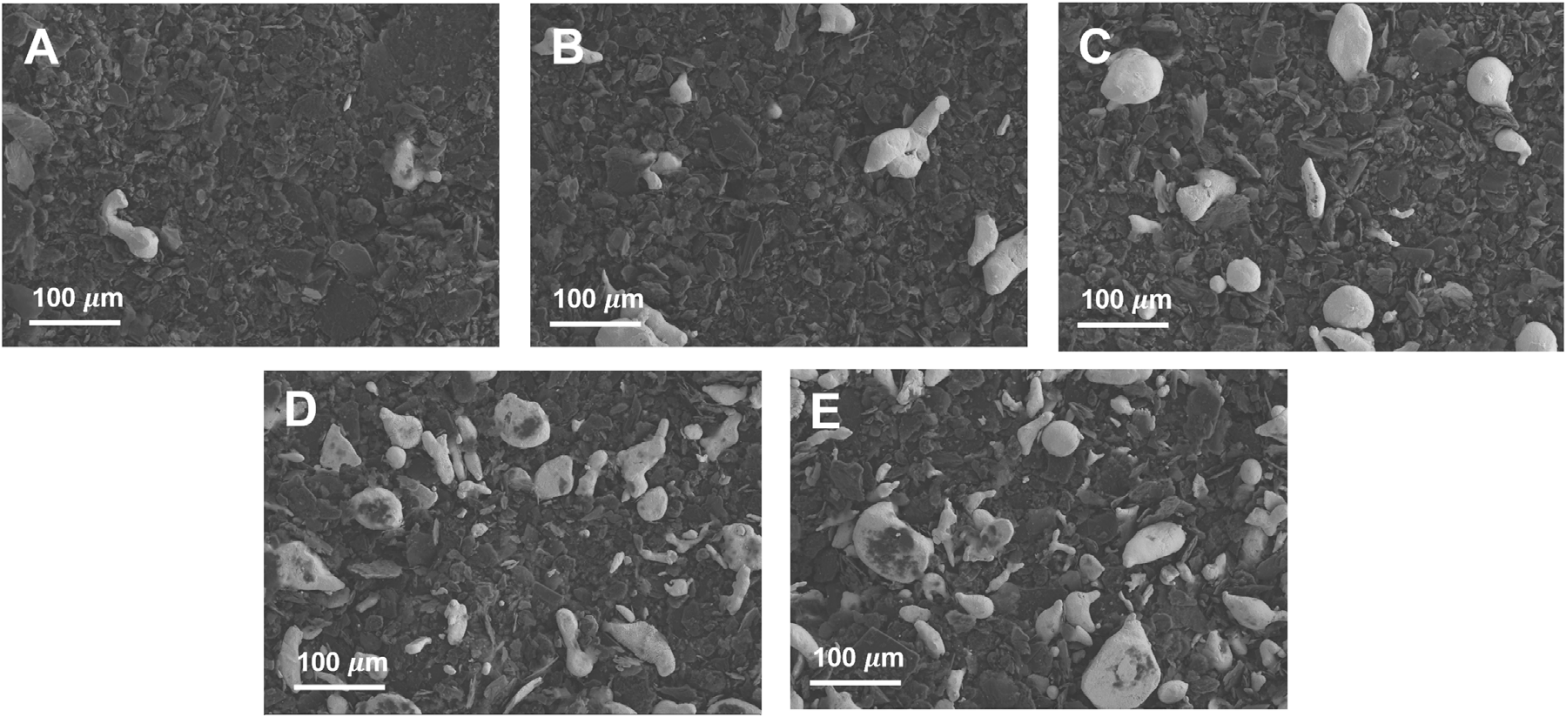
SEM surface analysis of Pb/C powders for **(A)** 1:8, **(B)** 1:4, **(C)** 1:1, **(D)** 4:1 and **(E)** 8:1 ratios.

Figures 1B–F shows the XPS analysis results of Pb/C powders with varied lead: carbon ratios. The Pb 4f spectra of Pb/C powders shows the Pb 4f_5/2_ and Pb 4f_7/2_ peaks of lead oxide at 142.7 eV and 137.8 eV, respectively. The intensity of the Pb peaks increased with the increase of the percentage of Pb. In particular, the Figure 1D of Pb/C 1:1 ratio showed dominant Pb metallic characteristic with a Pb metal peak around 137.0 eV. Based on the XPS peaks and intensities, we found that the higher the percentage of lead mixed in the powder, the more lead oxide was formed. The photographic images of the of fabricated bipolar plates with respect to the Pb:C ratio were displayed in Figures 2A–E. Furthermore, the scanning electron microscopy (SEM) images of prepared bipolar plates with different Pb and C ratios were analyzed. Although the Pb peaks of 1:4 and 1:8 ratios were barely detectable in XRD patterns, the respective Pb particles were found within the carbon with the size of approximately 50–70 μm, as shown in Figures 3A,B. One could see the presence of Pb as white particles in the SEM images, which becomes more prominent as the Pb content increases. As shown in Figure 3C, the Pb particles grew into more spherical particles in the Pb and C ratio of 1:1. With the high content of Pb in 4:1 and 8:1 (Figures 3D, E), the particles were more embedded in the carbon matrix, and the sizes varied in a wide range from 10 μm to 100 μm.

**FIGURE 4:**
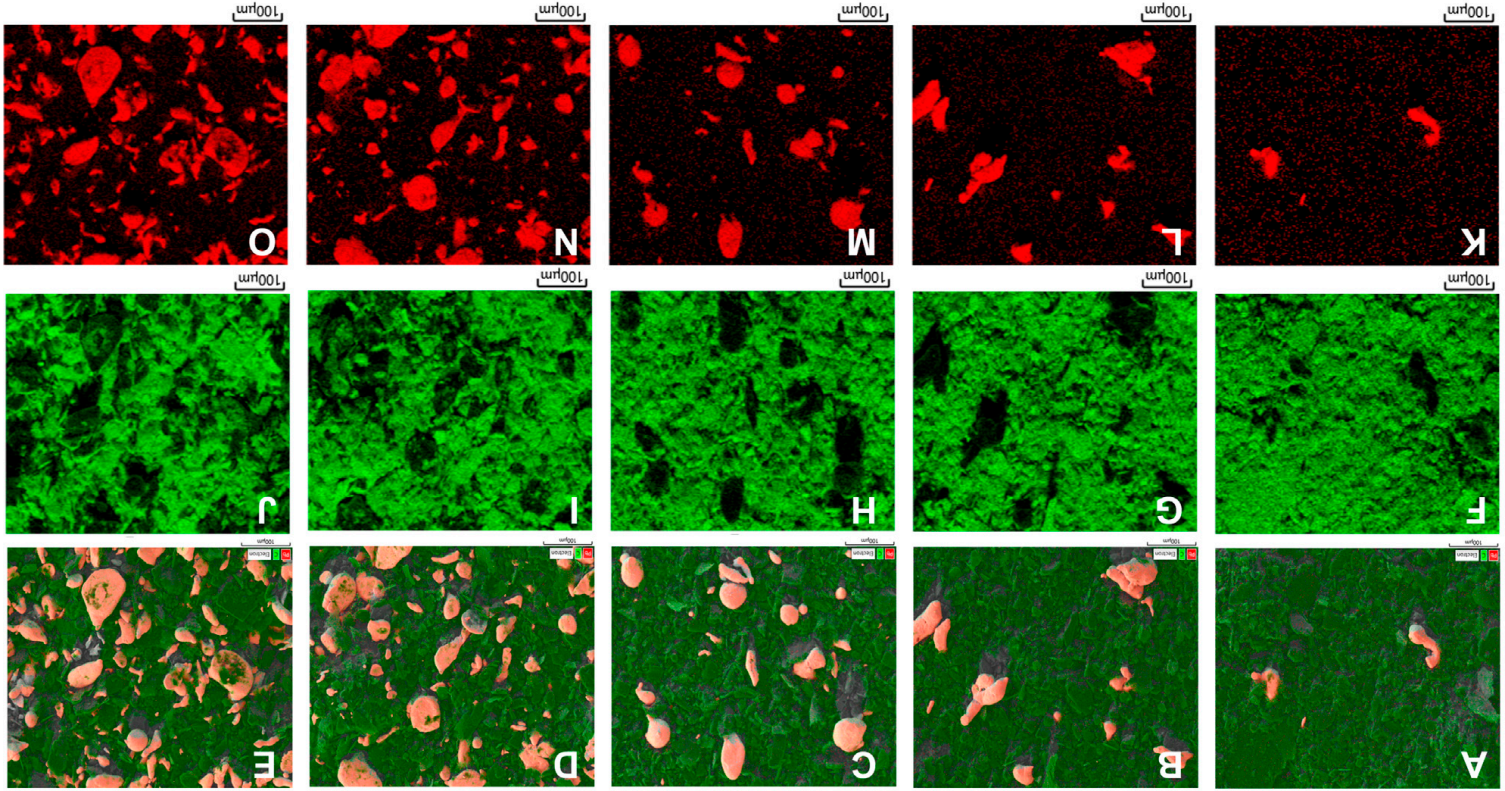
Elemental mapping of Pb/C powder in **(A)** 1:8, **(B)** 1:4, **(C)** 1:1, **(D)** 4:1 and **(E)** 8:1 ratios; C element **(F)** 1:8, **(G)** 1:4, **(H)** 1:1, **(I)** 4:1 and **(J)** 8:1 ratios; Pb element **(K)** 1:8, **(L)** 1:4, **(M)** 1:1, **(N)** 4:1 and **(O)** 8:1 ratios.

**FIGURE 5:**
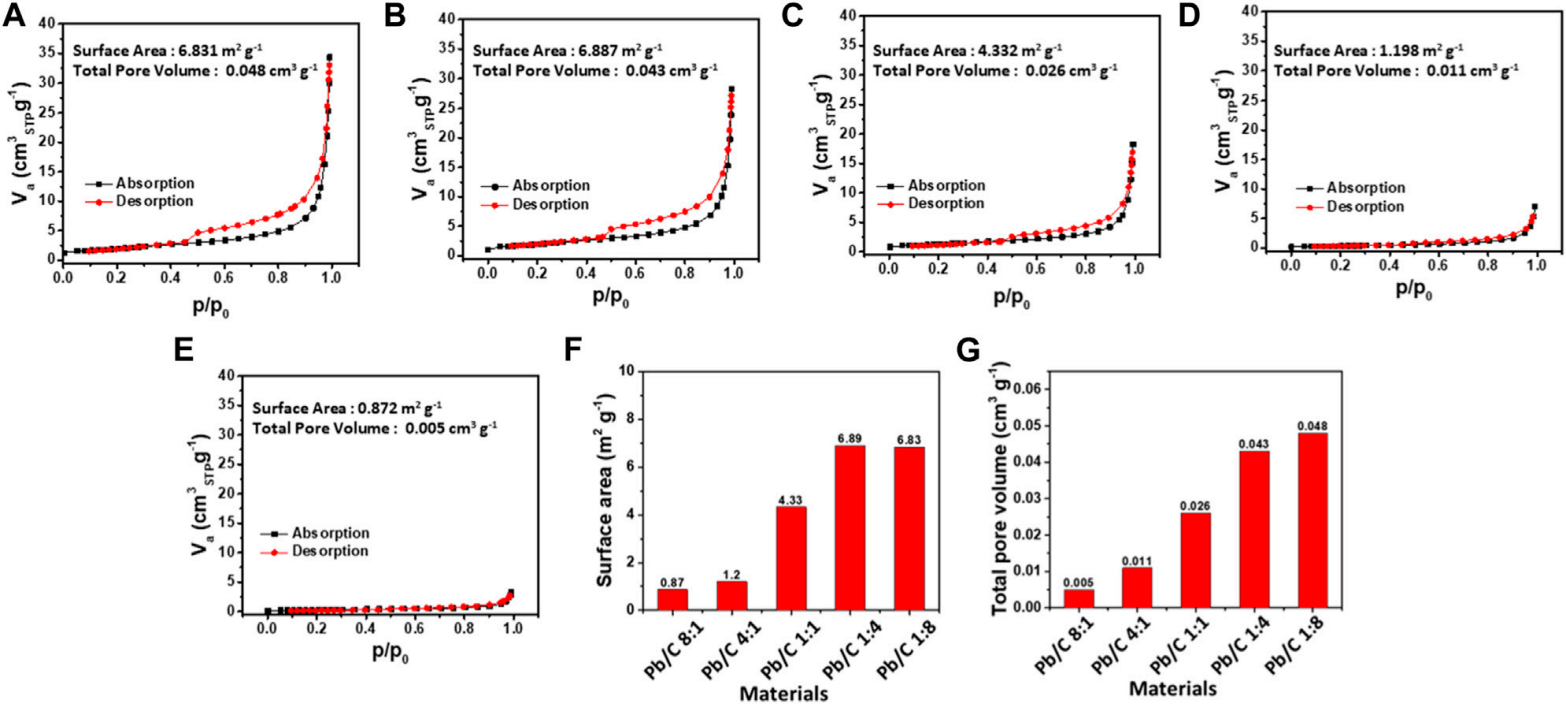
N_2_ adsorption and desorption of different ratios of Pb/C powder materials; **(A)** 1:8, **(B)** 1:4, **(C)** 1:1, **(D)** 4:1 and **(E)** 8:1 ratios; **(F)** Specific surface area and **(G)** total pore volume comparisons.

In Figure 4, the elemental mapping of the SEM images of Pb/C powder according to the mixing ratio was measured. In Figures 4A–E, the Pb particles are sufficiently and evenly dispersed through the ball mill process for Pb/C powder, and the number of particles in the image increases with the proportion of Pb with even distribution. In Figures 4F–J; Figures 5K–O, we can see the separate single element mapping images of carbon and lead, respectively.

### 3.2 Specific surface area and porosity

The Brunauer-Emmett-Teller (BET) analysis was done with N_2_ gas adsorption to measure the specific surface area and the pore volume. The specific surface area of each Pb/C bipolar plate was measured as shown in Figures 5A–G. With the high ratio of C, the specific surface area of 1:8 and 1:4 ratio plates were as large as 6.831 m^2^g^-1^ and 6.887 m^2^g^-1^, respectively. A slight decrease was shown in the 1:1 plate with a specific surface area of 4.332 m^2^ g^-1^. However, the high Pb ratio, 4:1 and 8:1 plates, showed a great decrease to 1.198 m^2^g^-1^ and 0.872 m^2^g^-1^, respectively. The total pore volume of the Pb/C plates shows similar trends to the specific surface area measurement results, where the 1:8 ratio of Pb to C showed the highest pore volume of 0.048 cm^3^g^-1^. Subsequently, the 1:4 ratio plate shows a higher pore volume of 0.043 cm^3^ g^-1^ which is due to the less dense carbon property compared to much denser Pb metal. Following the trend, the total pore volumes were measured as 0.026 cm^3^g^-1^ (1:1 ratio), 0.011 cm^3^g^-1^ (4:1 ratio), and 0.006 cm^3^ g^-1^ (8:1 ratio). The BET analysis shows that the higher content of Pb in the 8:1 and 4:1 ratio possesses more metallic alloy-like properties, resulting in much lower pore volumes and a decrease in exposed surface areas. On the other hand, as the C ratio increases from 1:1 to 1:4 and 1:8, the pore volume tends to increase, thereby, the surface area increases. The carbon mixed into the lead lowers the density of the plates for lightweight utilizations.

### 3.3 Physicochemical analysis

**FIGURE 6:**
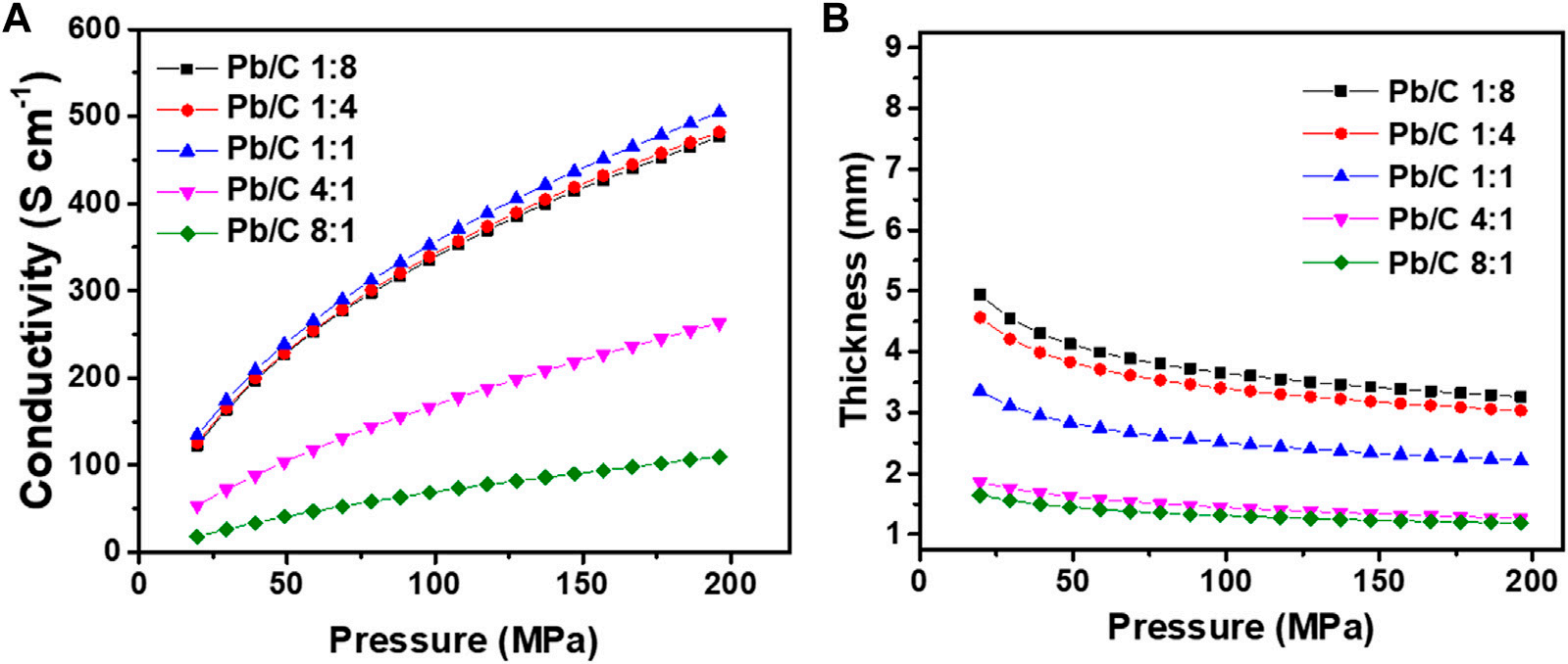
Changes in **(A)** electrical conductivity and **(B)** thickness of Pb/C composite powder according to changes in pressure.

The changes in electrical conductivity and the thickness under pressing pressure were measured as shown in Figure 6. At the low pressure of 20 MPa (Figure 6A), the electrical conductivity of the plate with the lead carbon ratio of 1:1 showed the highest conductivity of 133.94 S cm^−1^ compared to the 1:4 and 1:8 ratio plates with 125.67 S cm^−1^ and 122.48 S cm^−1^, respectively. On the other hand, where the Pb content increases, an enormous decrease in conductivity was measured as low as 53.76 S cm^-1^ (4:1 ratio) and 18.04 S cm^-1^ (8:1 ratio). These results indicate that carbon has a greater effect on conductivity than lead. As the pressure increases, the conductivity of the plates increases overall. The conductivity of the 1:1 ratio plate was increased by approximately 3.77 folds from 133.94 S cm^-1^–504.40 S cm^-1^, which was the highest conductivity among the synthesized bipolar plates at high pressure of 200 MPa.

The conductivity of the 1:4 and 1:8 ratio plates was also greatly increased to 481.99 S cm^-1^ and 476.33 S cm^-1^, respectively. The plates with higher Pb content have only increased by 91.61 S cm^-1^ for the 4:1 ratio (to 263.03 S cm^-1^ at 200 MPa) and 209.27 S cm^-1^ for the 8:1 ratio (to 109.65 S cm^-1^ at 200 MPa). Figure 6B shows the changes in the thickness of the synthesized bipolar plates under the pressing pressure of 20 MPa–200 MPa. The initial thickness of the plates showed the tendency to decrease as the Pb ratio increased, which may be due to an increase in the total pore volume corresponding with the BET results in Figure 5. The plate with the 1:8 ratio of Pb and C was the thickest with approximately 4.94 mm under 20 MPa of pressing pressure. The plates with other ratios were measured as 4.56 mm (1:4 ratio), 3.56 mm (1:1 ratio), 1.86 mm (4:1 ratio), and 1.65 mm (8:1 ratio). These tendencies correlate with the total pore volumes where the carbon opens the pores within the plates for increased surface area and decreased density. Correspondingly, when the pressure increases to 200 MPa, there is a huge drop in thickness. The thickness of the plates of 1:8, 1:4, and 1:1 ratio gets decreased to 3.26 mm, 3.03 mm, and 1.34 mm, respectively. The high Pb-contented plates showed minor changes in the thickness of 0.60 mm (4:1 ratio) and 0.46 mm (8:1 ratio) due to low pore volumes in the plates.

**FIGURE 7:**
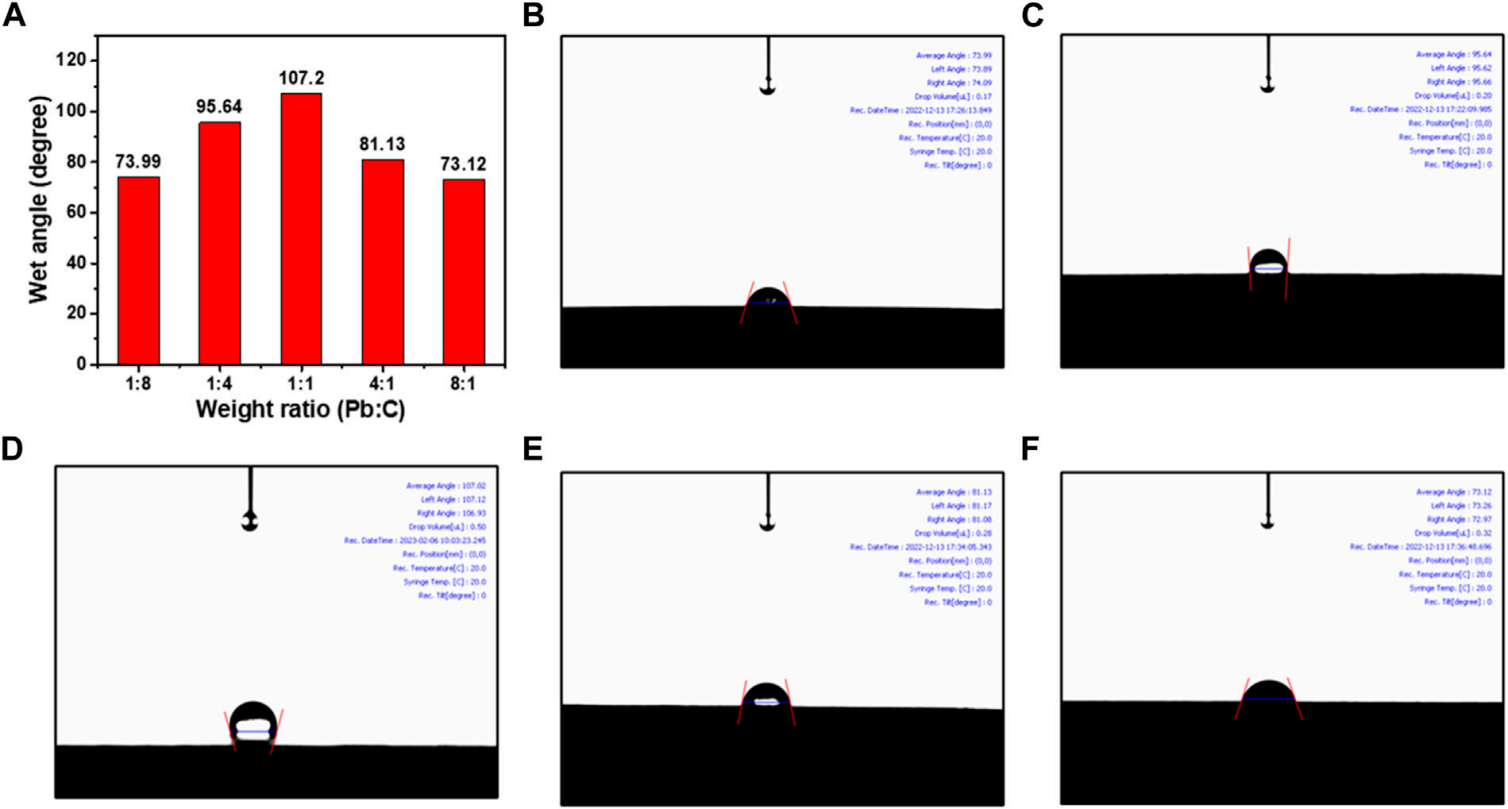
Water contact angle measurement results; **(A)** comparison table, Pb/C bipolar plates with ratios of **(B)** 1:8, **(C)** 1:4, **(D)** 1:1, **(E)** 4:1, **(F)** 8:1.

As shown in Figures 7A–F, the water contact angle measurement was taken to investigate the wettability properties of the synthesized bipolar plates. The wettability indicates the hydrophilicity of the bipolar plates, which can greatly affect the transportation of the electrolyte and the resultant water molecules from the URFC (Ait Djafer et al., 2014). The inner contact angle of the water droplet was measured to identify the hydrophilicity where the 1:1 ratio plate was the highest with an angle of 107.20°. The high angle of inner contact angle indicates that the plate is more hydrophobic, allowing for better transport of the electrolytes and resultant water molecules from URFC. The result also indicates that the 1:1 ratio plate was synthesized much more densely, and the roughness of the surface is distributed evenly. The contact angle of the plate with 1:4 and 4:1 ratio was measured as 95.64° and 81.13, respectively. The droplet contact angle decreases when either the Pb or C content in the prepared lead carbon ratios becomes more biased. Both plates with the 1:8 ratio and 8:1 ratio show much lower contact angles of 73.99° and 73.12, respectively, which indicates that the plates are more hydrophilic compared to the 1:1 ratio of Pb and C. Considering the electrical conductivity under the pressing pressure and the wettability properties, the Pb/C bipolar plates, synthesized with a 1:1 ratio, are best suited for utilization in URFC. The plate with a 1:1 ratio of Pb and C also shows moderate effects in thickness under pressing pressure, indicating that the plate can withstand the pressure when stacked in URFC.

### 3.4 Electrochemical analysis

**FIGURE 8:**
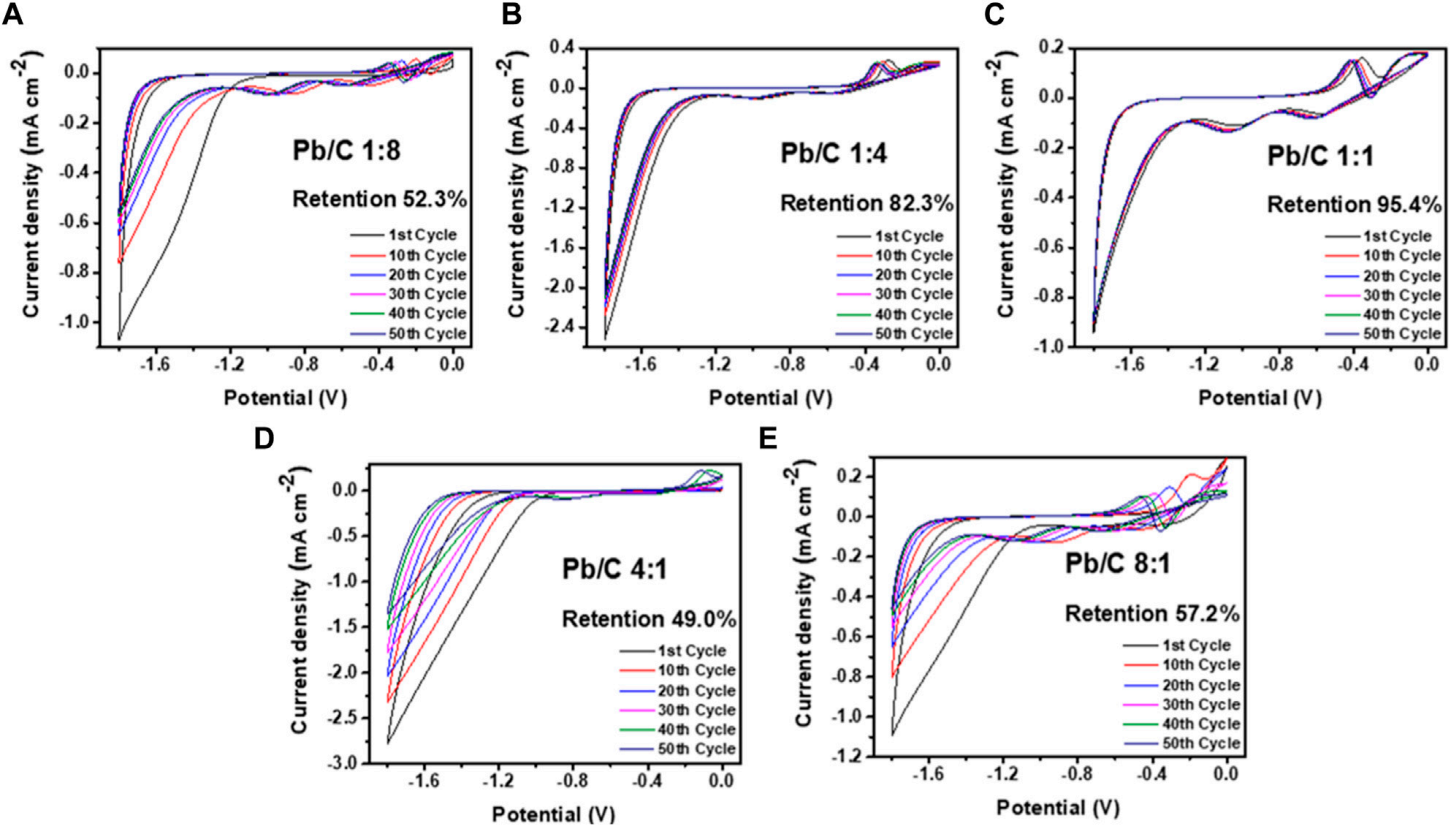
CV analysis using negative potentials in acidic medium (0.5 M H_2_SO_4_) at scan rate of 50 mV/sec; **(A)** 1:8, **(B)** 1:4, **(C)** 1:1, **(D)** 4:1, and **(E)** 8:1 ratios.

**FIGURE 9:**
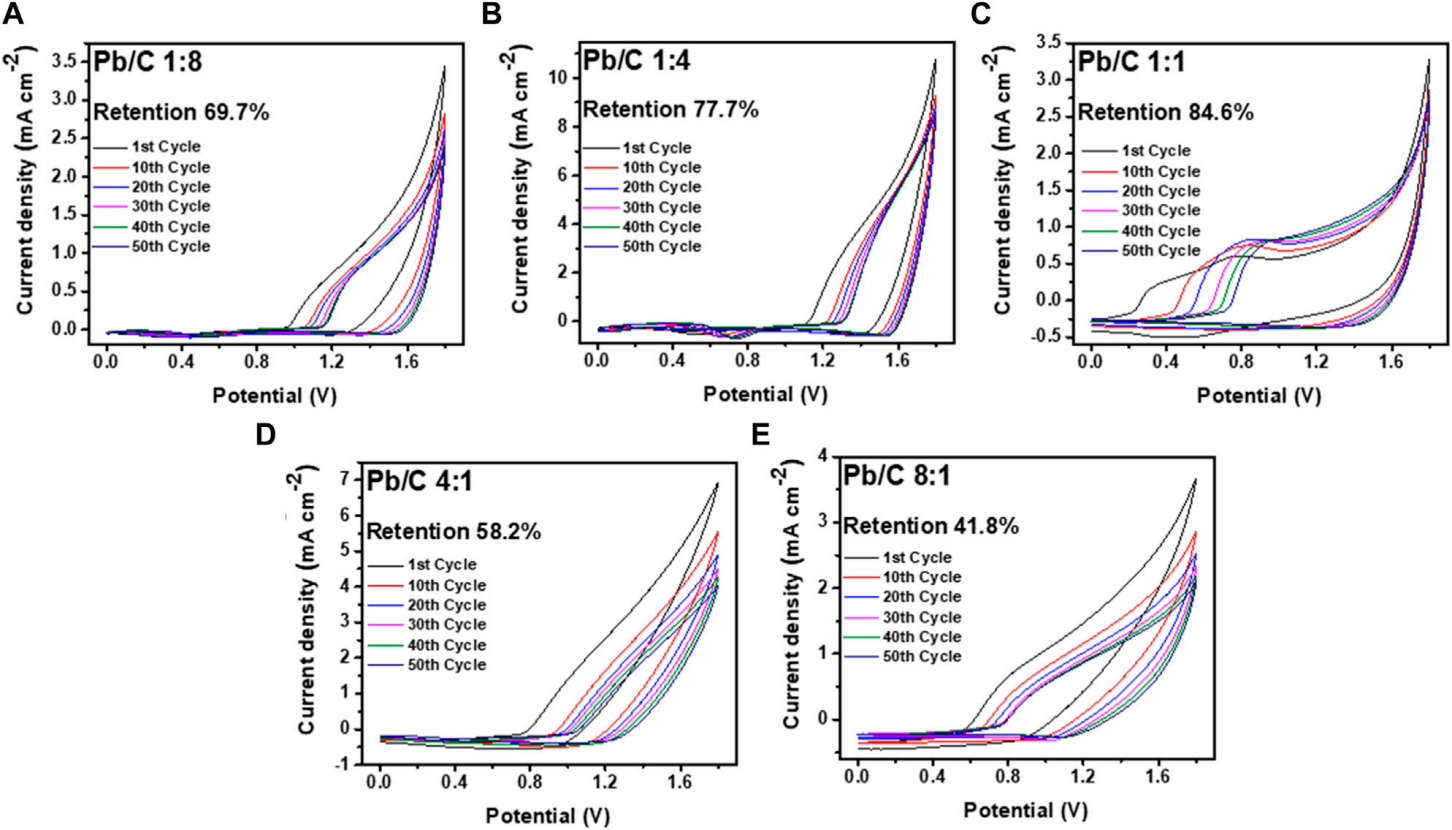
CV analysis using positive potentials in acidic medium (0.5 M H_2_SO_4_) at scan rate of 50 mV/sec; **(A)** 1:8, **(B)** 1:4, **(C)** 1:1, **(D)** 4:1, and **(E)** 8:1 Pb/C ratios.

The electrochemical retention rate was measured to investigate the electrochemical capability through multiple uses for URFCs. Cyclic voltammetry was measured in negative and positive potential ranges, as shown in Figures 8A–E; Figures 9A–E. Each plate was run through 50 cycles in the acidic medium of 0.5 M H_2_SO_4_ electrolyte in the three-electrode configuration with Pt wire and Ag/AgCl electrode as counter and reference electrodes, respectively. The CV analysis shows the percentage decrease in the current density value at 1.8V compared to the first and 50th cycle results as the retention rate.

Measurement through the negative potential range of - 1.8 V–0.0 V vs. Ag/AgCl, shows that the Pb/C plate with a 1:1 ratio had the highest stability with a 95.4% retention rate over 50 CV cycles. The Pb to C ratio of the 1:1 plate showed electrochemically most stable performances indicating that it is most suitable for URFC bipolar plate. Subsequently, the 1:4 ratio plate shows better retention, where approximately 82.3% of the first performance was maintained after multiple cycles. The plates with biased Pb or C content showed almost half the retention rate of the 1:1 plate. Both the 1:8 and 8:1 plates showed a retention rate of 52.3% and 57.2%, respectively, whereas the 4:1 plate showed the lowest retention rate of 49.0% after the 50th cycle. The great decrease in retention rate indicates that an even ratio of Pb and C is more electrochemically stable in the negative potentials. The positive potential range of 0 V–1.8 V vs. Ag/AgCl was measured with the same configuration, as shown in Figure 9. Similar to the negative potential, the 1:1 ratio of Pb and C plate showed the highest retention rate over 50 cycles with the rate of 84.6%, followed by 1:4 ratio plate with 77.7% retention. The high Pb ratios of 4:1 and 8:1 also showed a poor retention rate in the positive potentials, with rates of 58.2% and 41.8%, respectively. Comparatively, the 1:8 plate, which showed the lowest retention rate in the negative potentials, had a retention rate of 69.7% which was higher than that of the 4:1 and 8:1 ratios.

**FIGURE 10:**
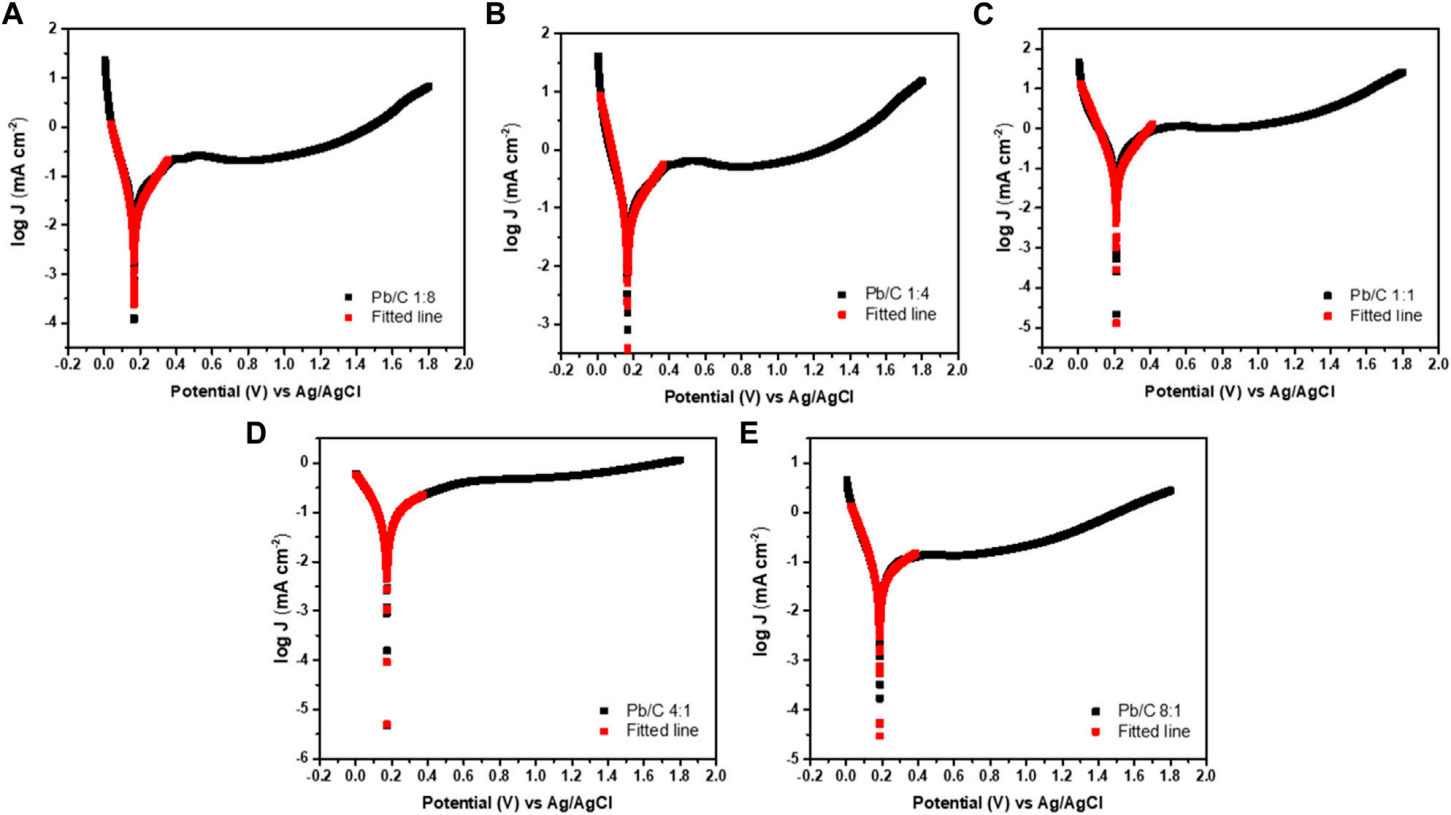
Potentiodynamic polarization curves in acidic medium (0.5 M H_2_SO_4_); **(A)**1:8, **(B)** 1:4, **(C)**1:1, **(D)**4:1, and **(E)** 8:1 ratios at the scan rate of 50 mV/sec.

Figure 10 shows the potentiodynamic polarization curves of the Pb/C bipolar plate with different compositions of 1:8, 1:4, 1:1, 4:1, and 8:1 ratios in the simulated 0.5 M H_2_SO_4_ solution. In the range of 0–1.8V (vs. Ag/AgCl), the corrosion potential of Pb/C 1:8, 1:4, 1:1, 4:1, and 8:1 is 0.165 V, 0.167 V, 0.211 V, 0.173 V, and 0.185 V. Thermodynamically, the higher the corrosion potential, the higher the potential must be applied for the reaction to occur. In other words, it has high chemical inertness and corrosion resistance (Zhao et al., 2016). Among the candidates, the corrosion potential of the Pb/C 1:1 ratio was the highest, indicating that the corrosion resistance of the composite separator can be improved if Pb and carbon are combined in an appropriate ratio.

The overall electrochemical analysis indicates that the Pb and C ratio of 1:1 is best suitable for the utilization in URFC as a bipolar plate due to its high durability over multiple cycles. As the ratio of one element increases to the other, the durability tends to degrade in acidic media. The electrical properties under pressing pressure and the electrochemical analysis show that employing the most efficient 1:1 ratio Pb/C bipolar plate will possibly serve the highly efficient electrochemical system, the URFC.

From the analyzed results, it was found that the pressed density, porosity, electrical conductivity, electrochemical durability, and corrosion resistance are different depending on the ratio of carbon and lead and that the properties tend to decrease when the ratio of either carbon or lead increases. This means that bipolar plates made of mixed Pb/C powder and lead below the melting point have bulk properties due to the presence of bulk particles. The distance between particles, porosity between particles, and the degree of grain boundary formation varies depending on the composite powder ratio, which affects electrical conductivity (Khodabakhshi et al., 2020). It also affects the compaction rate and density. Since corrosion resistance increases in proportion to density, increasing the proportion of lead with fewer pores and higher density than carbon improves the corrosion resistance of bipolar plates (Singh et al., 2019). In Figure 1D, XPS spectra of Pb/C powder at a 1:1 ratio show that the Pb metallic characteristic dominates the Pb metallic peak of Pb/C powder at a 1:1 ratio, showing comprehensive superiority in physical and electrochemical properties over other ratios, which are dominated by lead oxide peaks.

## 4 Conclusion

In this work, various proportions of lead/carbon composite materials are manufactured by a simple heating-compression process using SBR as a binder, and the electrochemical properties of the prepared bipolar plates are studied for being employed as separators in URFC. The morphological features, surface chemical nature, porosity, specific surface area, conductivity upon compressibility, wettability, and electrochemical performance of the varied proportions of lead carbon composite-based bipolar plates are examined in detail through essential physicochemical and electrochemical characterization techniques. The 1:1 ratio of lead/carbon composites showed better stability and high conductivity among all the prepared variants. The porosity and electrical conductivity of the composite are observed to increase with the increased proportion of carbon. The 1:1 ratio showed high hydrophobicity through a high contact angle, and its highest retention rate during electrochemical analysis ensures the durability of the prepared bipolar plate. Based on these results, we confirmed the possibility of a lead/carbon composite-based to be employed as a separator to URFC and could be a candidate for a new separator for URFC. It will also contribute to the development of separators that can be used not only in URFC but also in various applications.

## Data Availability

The raw data supporting the conclusion of this article will be made available by the authors, without undue reservation.
